# Beyond Antibiotics: Therapeutic Strategies Utilizing Probiotics and Bacteriophages Against Drug-Resistant *Staphylococcus aureus*

**DOI:** 10.3390/microorganisms14020344

**Published:** 2026-02-02

**Authors:** Miao Zhao, Dongli Rong, Ling Chen, Shuzhen Cai, Yongcheng Lu, Jingsha Dai, Youxiong Zhang, Xianhu Wei, Xiaojuan Yang, Qingping Wu

**Affiliations:** 1 School of Food and Biological Engineering, Shaanxi University of Science and Technology, Xi’an 710021, China; zm93032@163.com; 2 State Key Laboratory of Applied Microbiology Southern China, Guangdong Provincial Key Laboratory of Microbial Safety and Health, National Health Commission Science and Technology Innovation Platform for Nutrition and Safety of Microbial Food, Key Laboratory of Big Data Technologies for Food Microbiological Safety, State Administration for Market Regulation, Institute of Microbiology, Guangdong Academy of Sciences, Guangzhou 510070, China; rongdl69@126.com (D.R.); chenling@gdim.cn (L.C.); shuzhen908@163.com (S.C.); luyongcheng@gdim.cn (Y.L.); daijs@gdim.cn (J.D.); zyx0713141061@126.com (Y.Z.); wxhu7508@163.com (X.W.); 3 Food and Drug Laboratory, Guangdong Detection Center of Microbiology, Guangzhou 510070, China

**Keywords:** *Staphylococcus aureus*, antibiotic resistance, probiotics, bacteriophage, lysins, biocontrol strategies

## Abstract

*Staphylococcus aureus*, as a critical zoonotic and foodborne pathogen, not only triggers public health threats such as food poisoning but also acts as a leading cause of diverse clinical infections, including skin infections, pneumonia, and endocarditis. Confronted with the growing crisis of multidrug resistance in *S. aureus*, both phage therapy and probiotic therapy have emerged as promising alternative biological strategies; however, the current literature predominantly examines them in isolation. This review therefore aims to delineate the contemporary therapeutic challenges of drug-resistant *S. aureus* and to systematically compare the mechanisms and clinical translation of phages and probiotics within an integrated analytical framework. We first outline the current therapy landscape, then present side by side the molecular inhibitory mechanisms and clinical progress of both approaches, followed by a comparative analysis of their antibacterial mechanisms, clinical efficacy, and industrial challenges. Through this consolidated perspective, the review not only clarifies the distinct strengths and limitations of each strategy but also seeks to provide researchers and clinicians with a comprehensive mechanistic and evidence-based reference for developing novel antibacterial technologies and designing innovative therapeutic regimens. Ultimately, it highlights potential synergies between phages and probiotics, offering a forward-looking roadmap to overcome *S. aureus* resistance and advance personalized combinatorial therapies.

## 1. Introduction

*S. aureus* is ubiquitously distributed in natural environments and on the skin and nasal cavities of humans and animals. Although it is a common commensal bacterium, it possesses considerable pathogenicity [1]. It can cause a variety of infectious diseases, including skin and soft tissue infections, pneumonia, endocarditis, osteomyelitis, foodborne intoxication, and bacteremia [2], posing a serious threat to immunocompromised individuals in particular, even endangering their lives [3]. As one of the deadliest types of *S. aureus* infection, bacteremia has a mortality rate of 15–30%, accounting for approximately 300,000 deaths worldwide each year [4]. At the same time, the crisis of antibiotic resistance caused by antibiotic overuse is pushing modern medicine into the abyss of “no drugs available” [5]. In recent years, the problem of resistance of *S. aureus* to multiple antibiotics has continued to worsen, with the emergence of methicillin-resistant (MRSA), vancomycin-intermediate resistance (VISA), and vancomycin-resistant (VRSA) strains, as well as resistance to novel antibiotics such as daptomycin and linezolid; furthermore, the formation of tolerant and persistent bacteria further impairs therapeutic effectiveness, leading to an increasing scarcity of clinical therapeutic options and posing a serious threat to global public health security [6]. The latest study in The Lancet [7] predicts that the number of deaths caused by antibiotic resistance worldwide may exceed 39 million in the next 25 years.

The severity of the above issues highlights the urgent need for innovative therapeutic approaches. In recent years, green biocontrol strategies such as probiotics and bacteriophages have exhibited substantial potential in the field of anti-drug-resistant *S. aureus*. Unlike antibiotics, probiotics can effectively inhibit the colonization and infection of *S. aureus* through multiple mechanisms, such as competitive exclusion, secretion of antimicrobial agents, and regulation of the host immune response [8,9]. Bacteriophages can efficiently eradicate drug-resistant strains by directly lysing bacteria, disrupting biofilms, regulating immunity, and inhibiting the expression of virulence factors [10]. Although there have been numerous studies on the antibacterial efficacy of both, and both are key research directions for the new generation of advanced antibacterial strategies [11], there is still a lack of systematic exploration that integrates the theoretical mechanisms and clinical applications of both under the same analytical framework. Therefore, such integrated research is of great necessity. Based on this, this review will systematically review the infection mechanisms and therapeutic challenges caused by *S. aureus* from the key perspective of clinical management of *S. aureus* infection, focusing on the research progress of the antibacterial mechanism and clinical therapeutic status of these two novel green biological control strategies.

The core value of this review lies in integrating the antibacterial mechanisms of probiotics and bacteriophages with current mainstream clinical therapeutic scenarios, closing the gap between basic research and clinical translation. At the same time, we conduct a systematic comparative analysis of the two from the perspectives of mechanisms, clinical applications, and implementation challenges, not only clarifying the core hurdles currently faced by the two in clinical practice but also proposing prospective therapeutic mechanisms for the future. This work can provide comprehensive references covering theoretical mechanisms and clinical applications for relevant personnel, laying a solid foundation for the development of new antibacterial strategies that transcend the traditional antibiotic paradigm.

## 2. Literature Search Strategy and Study Selection

This review was conducted in accordance with the general methodological guidelines of narrative reviews. The literature search comprehensively covered multiple databases such as PubMed, SpringerLink, Web of Science Core Collection, ResearchGate, ScienceDirect, and MDPI. We also manually supplemented the references of relevant literature and reviews. The search period was set from 2013 to 2026, aiming to capture data from approximately the past decade on: (1) the pathogenic mechanisms, drug resistance mechanisms, and therapeutic challenges of *S. aureus*; (2) the antibacterial mechanisms and clinical therapeutic status of probiotics; and (3) recent advances in the antibacterial mechanisms and clinical therapeutic status of bacteriophages. During the initial search, no restrictions were imposed on the country or research type.

We have formulated corresponding search strategies based on the characteristics of each database, and the search formulas mainly incorporate the following core terms: “virulence mechanism of *S. aureus*”, “resistance mechanism of *S. aureus*”, “therapeutic regimens for *S. aureus* infections”, “mechanism of probiotic inhibition of *S. aureus*”, “clinical therapy of *S. aureus* infection with probiotics”, “mechanism of phage inhibition of *S. aureus*”, and “clinical application of bacteriophages for *S. aureus* infections”.

## 3. *S. aureus*: Virulence Mechanisms, Antimicrobial Resistance Mechanisms, and Therapeutic Dilemmas

*S. aureus*, as a major human pathogen, presents significant challenges in clinical management. These challenges stem from its dual evolutionary strategies: on the one hand, it actively damages host tissue and triggers excessive immune responses by secreting various virulence factors such as pore-forming toxins and superantigens; on the other hand, through genetic acquisition and mutation, it has developed complex resistance mechanisms, including antibiotic-resistance genes (e.g., *mecA*) and biofilm formation, to withstand antimicrobial agents. Virulence and resistance mechanisms often act synergistically, rendering conventional antibiotic therapy not only ineffective but also potentially detrimental by inducing toxin release and exacerbating the infection.

### 3.1. Virulence Mechanisms

**Figure 1:**
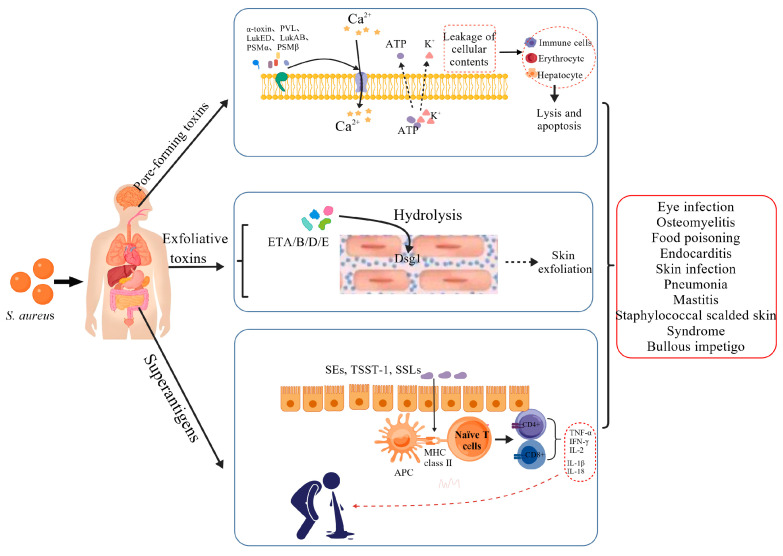
Pathogenic mechanisms of *S. aureus* virulence factors. (Created with BioGDP.com [27]). Notes: **Pore-forming toxins** (e.g., α-toxin, PVL, PSMα/β) create pores on host cell surfaces, leading to cell lysis, apoptosis, and ultimately tissue damage. **Exfoliative toxins** (e.g., ETA, ETB, ETD, ETE) primarily disrupt desmosomal adhesion by cleaving desmogleins between keratinocytes, resulting in epidermal detachment. **Superantigens**, particularly enterotoxins, bind nonspecifically to both MHC class II molecules on antigen-presenting cells and the Vβ region of T-cell receptors, directly activating T-cell clones and triggering a potent immune response in the host.

During the early stages of *S. aureus* infection, the pathogen initiates a multi-pronged assault by secreting a diverse array of virulence factors, triggering a cascade of events that progress from local tissue damage to a systemic inflammatory storm. The toxins produced by *S. aureus* are functionally categorized into three main classes: pore-forming toxins, exfoliative toxins, and superantigens, as shown in Figure 1.

#### 3.1.1. Pore-Forming Toxins (PFTs)

Pore-forming toxins primarily include α-toxin (α-hemolysin, Hla) [12], leukocidins such as Panton–Valentine leukocidin (PVL) [13], γ-hemolysin [14], LukED [15], and LukAB/GH [16], as well as phenol-soluble modulins (including PSMα, PSMβ, and δ-toxin (PSMγ)) [17]. A common feature of these toxins is their ability to recognize and bind specific receptors on host cell membranes, disrupt membrane integrity, and form pores, leading to the leakage of cellular contents (K^+^, Ca^2+^) and ultimately to the lysis of immune and other somatic cells, resulting in tissue damage—a key mechanism in virulence [18]. Notably, toxins like PVL are strongly associated with necrotizing pneumonia and severe skin infections caused by community-associated methicillin-resistant *S. aureus* (CA-MRSA) [13].

#### 3.1.2. Exfoliative Toxins

*S. aureus* produces exfoliative toxins (ETs), a group of serine proteases with high substrate specificity, whose core mechanism lies in the precise cleavage of key structural proteins in the host skin, leading to characteristic dermatological lesions. To date, five ETs have been identified: ETA, ETB, ETC, ETD, and ETE. Among them, ETA, ETB, and ETD specifically recognize and hydrolyze a conserved peptide bond within the extracellular domain of desmoglein-1 (Dsg1), a cadherin-type protein located in the desmosomes between epidermal keratinocytes; this cleavage disrupts intercellular adhesion, resulting in splitting within the epidermal granular layer and subsequent exfoliation, clinically manifested as staphylococcal scalded skin syndrome (SSSS) or bullous impetigo [19,20]. ETE specifically cleaves Dsg1 from sheep, goats, mice, and humans but shows no activity against bovine Dsg1 [21], while ETC, also known as adenylosuccinate lyase, exhibits a three-dimensional structure distinct from other ETs and is non-toxic to humans [22].

#### 3.1.3. Superantigens

The core pathogenic mechanism shared by *S. aureus* superantigens lies in their ability to act as “molecular bridges,” simultaneously and nonspecifically binding to major histocompatibility complex class II molecules on antigen-presenting cells and the Vβ variable region of T-cell receptors. This interaction bypasses conventional antigen processing and presentation, leading to massive polyclonal activation of up to 20% of T-cell clones. The resulting excessive immune response, often referred to as a “cytokine storm,” underlies acute life-threatening conditions such as toxic shock syndrome [23]. For example, toxic shock syndrome toxin-1 (TSST-1) can cause high fever, hypotension, and multiple organ failure [24]; staphylococcal enterotoxins (SEs) are a major cause of food poisoning [25]; and staphylococcal superantigen-like proteins (SSLs) interfere with the complement system or neutrophil function to facilitate bacterial immune evasion [26].

#### 3.1.4. Summary

*S. aureus* employs a sophisticated arsenal of virulence factors—pore-forming toxins, exfoliative toxins, and superantigens—to breach host barriers, damage tissues, and dysregulate immune responses. These mechanisms collectively enable the pathogen to establish infections ranging from localized skin lesions to life-threatening systemic syndromes. Understanding this multifactorial virulence landscape is crucial for developing therapies that not only target bacterial viability but also neutralize these pathogenic determinants.

### 3.2. Antimicrobial Resistance Mechanisms

*S. aureus*, particularly methicillin-resistant *S. aureus* (MRSA), exhibits multifaceted and intricate resistance mechanisms, encompassing both intrinsic and acquired pathways [28]. Intrinsic resistance refers to the innate defensive capabilities of the bacterium. The core mechanism involves the *mecA* gene, which encodes the penicillin-binding protein 2a (PBP2a) with very low affinity for β-lactam antibiotics, thereby rendering this drug class ineffective [29,30]. Additional inherent strategies include the production of β-lactamases that hydrolyze antibiotics in synergy with PBP2a [31,32]; activation of efflux pumps (e.g., NorA, NorB, NorC, QacA) that reduce intracellular drug concentrations [33,34,35]; thickening of the cell membrane and cell wall to hinder drug penetration; and reliance on accessory gene regulators involved in cell wall metabolism, as observed in heterogeneous vancomycin-intermediate *S. aureus* (hVISA) and vancomycin-intermediate *S. aureus* (VISA) strains [36,37]. Acquired resistance mechanisms are those gained through horizontal gene transfer or mutation. MRSA can develop resistance via antibiotic target modification (e.g., point mutations in the 23S rRNA gene leading to linezolid resistance) [38,39]; acquisition of the *vanA* gene (originating from vancomycin-resistant enterococci) resulting in vancomycin-resistant *S. aureus* (VRSA) [40]; and biofilm formation, which shields bacteria from antimicrobial agents and promotes persistent infections [41,42].

In summary, the antimicrobial resistance of *S. aureus*, particularly MRSA, is a multifactorial and dynamic phenomenon involving both intrinsic (e.g., *mecA*-mediated PBP2a production, efflux pumps) and acquired (e.g., gene transfer, target modification, biofilm formation) mechanisms. This complex resistance network not only renders conventional antibiotics ineffective but also continuously evolves, posing a persistent challenge to clinical therapy and the urgent need for alternative therapeutic strategies.

### 3.3. Clinical Challenges in Treating S. aureus Infections

Based on the preceding literature review of pathogenic and resistance mechanisms, a comparative analysis indicates that the clinical therapy of *S. aureus* infections is becoming increasingly challenging due to the involvement of a wide array of virulence factors and complex, continuously evolving resistance mechanisms. The following section outlines the key dilemmas currently encountered in the clinical management of *S. aureus* infections.

In the therapy of *S. aureus* infections, particularly those caused by MRSA, current drugs and strategies face numerous limitations. Vancomycin, which inhibits cell wall synthesis, was once considered the gold standard for therapy, yet its application is constrained by two main challenges. The most prominent issue is the evolution of resistance—the emergence of vancomycin-intermediate (VISA) and vancomycin-resistant (VRSA) strains has significantly compromised its efficacy [43]. Additionally, vancomycin requires intravenous administration and carries risks of nephrotoxicity and ototoxicity [44], necessitating therapeutic drug monitoring, which further limits its clinical utility. Commonly used alternatives also have their constraints: linezolid, although orally available, is associated with side effects such as myelosuppression [45]; daptomycin is effective against bacteremia but is contraindicated in pneumonia and may exhibit cross-resistance with vancomycin [46,47]. Oral agents, such as sulfamethoxazole/trimethoprim (SMX/TMP) and doxycycline, are challenged by rapidly developing resistance [48]. Combination therapy strategies—such as vancomycin plus a β-lactam [49] or daptomycin plus a β-lactam [50]—are often employed to enhance efficacy, yet they still require individualized selection and vigilant monitoring for adverse effects and resistance risks. At a deeper level, therapeutic challenges persist due to several factors: biofilm formation, which impedes drug penetration [51]; the fact that traditional antibiotics primarily kill bacteria rather than inhibit toxin release, potentially allowing clinical symptoms to persist [52]; and heterogeneous resistance within bacterial populations, which may lead standard susceptibility tests to underestimate the actual level of resistance, thereby increasing the risk of therapy failure and recurrence [53].

Regarding the therapeutic challenges associated with various inflammatory diseases caused by *S. aureus* infections (such as conjunctivitis and mastitis), this literature review summarizes them, with specific results presented in Table 1.

**Table 1:** *S. aureus* Infection Types and Therapeutic Challenges.

Infection Type	Therapeutic Challenges
Ocular infection (conjunctivitis/keratitis)	1. Lack of topical-specific susceptibility criteria [54]; 2. Rising resistance (e.g., to fluoroquinolones, MRSA) [55]; 3. Biofilm-mediated therapeutic failure [56]; 4. Geographic variability in resistance [57]
Skin and soft tissue infection	1. High mortality [58]; 2. MRSA limits empirical therapy [59]; 3. Complex decision-making [60,61]; 4. Limited outpatient evidence [62]; 5. Therapeutic dilemma in drug selection [63]; 6. New drug approval uncertainty [64]
Pneumonia	1. Poor lung tissue penetration [65]; 2. Limited therapeutic drug monitoring (TDM) implementation [66]; 3. Drug toxicity with prolonged use [67]; 4. Lack of TDM data for newer antibiotics [68]
Endocarditis	1. MRSA infections and vancomycin resistance [69]; 2. Prosthetic valve/device-related infections [70]; 3. Outpatient IV/oral step-down therapy adherence and monitoring [71]; 4. Optimal timing of early surgery (<7 days) [72]
Osteomyelitis	1. Poor antibiotic penetration and MRSA resistance [73]; 2. Intracellular persistence and relapse [74]; 3. Physical barrier to drugs and immune cells [75]; 4. Surgical inaccessibility and recurrence [76]; 5. Immune suppression and chronicity [77]
Food poisoning	1. SEs are heat-stable [78]; 2. Limitations of traditional control [79]; 3. Antibiotic resistance [80]; 4. Biofilm persistence [81]; 5. Need for integrated strategies [82]
Bacteremia	1. Rising MRSA prevalence [83]; 2. Persistent and recurrent bacteremia [84,85]; 3. Limited benefit of combination therapy [50]; 4. Drug resistance and adverse drug reactions [86]
Mastitis	1. Antimicrobial resistance (AMR) [87,88]; 2. Biofilm-associated therapy failure [89]; 3. Difficulty eradicating intracellular bacteria [90,91]; 4. Therapy costs and low cure rates [92]

The compiled data in the table indicate that for most diseases caused by *S. aureus* infections, current clinical therapies are either constrained by antibiotic resistance issues or limited by antibiotic toxicity. These limitations collectively underscore the inadequacy of relying solely on conventional antibiotic-based therapeutic approaches and urgently signal the need to develop novel therapeutic strategies targeting virulence factors, biofilms, or host immune modulation.

## 4. Advances in Research on Probiotics Against *S. aureus*

As emphasized earlier, there is a pressing need to identify novel alternative or adjunct therapeutic strategies. Probiotics, as a promising biotherapeutic approach, inhibit *S. aureus* through multiple mechanisms and have demonstrated therapeutic potential across various infection models.

### 4.1. Mechanisms of Probiotics in Inhibiting S. aureus

**Figure 2:**
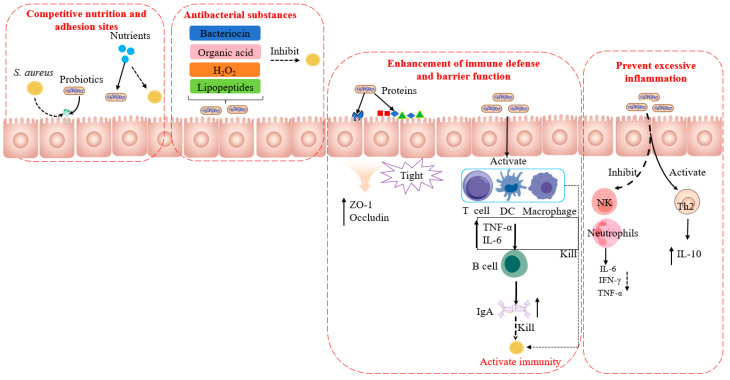
Mechanisms of probiotic-mediated antagonism against *S. aureus*. (Created with BioGDP.com [27] and Microsoft PowerPoint). Notes: This figure illustrates four main antagonistic strategies: **Competition exclusion:** probiotics restrict the survival of *S. aureus* by competing for intestinal nutrients and binding sites. **Secretion of antimicrobial compounds**: probiotics antagonize *S. aureus* by producing antimicrobial peptides, organic acids, surfactants, and hydrogen peroxide. **Enhancement of intestinal barrier and host immunity**: probiotics activate host immune cells to secrete inflammatory cytokines and IgA antibodies, thereby eliminating *S. aureus*. **Immunomodulation**: probiotics help balance excessive immune responses and prevent collateral tissue damage by promoting the secretion of the anti-inflammatory cytokine IL-10. Black solid arrows indicate binding, upregulation, or activation. Black dashed arrows represent failure to bind, downregulation, or inhibition.

Probiotics mainly inhibit *S. aureus* through three mechanisms, as shown in Figure 2.

#### 4.1.1. Competitive Exclusion

The competitive exclusion mechanism refers to an important antibacterial strategy whereby probiotics restrict the adhesion, colonization, and growth of *S. aureus* (particularly MRSA) by either occupying adhesion sites or competing for limited nutrients in shared environments. This mechanism primarily involves the following two aspects:

1. Adhesion Site Competition

Probiotics utilize various surface adhesins, such as proteins, lipoteichoic acids, and exopolysaccharides, to specifically bind to host epithelial receptors like mucins and integrins. This allows them to pre-emptively occupy adhesion sites, forming a physical barrier that blocks pathogen colonization [93,94]. Through this robust adhesion mechanism, probiotics can preferentially occupy ecological niches at different host sites and even on abiotic surfaces, thereby inhibiting *S. aureus*. For instance, *Lactobacillus plantarum* can competitively bind to epithelial cells in the urinary or intestinal tract via its surface proteins (e.g., GAPDH), effectively impeding the adhesion of *S. aureus* [95,96]. Furthermore, extracellular polymeric substances secreted by probiotics, including polysaccharides, proteins, and DNA, contribute to biofilm formation, further enhancing their attachment stability and ecological dominance on host surfaces [97].

2. Nutrient Competition

Probiotics compete with MRSA for limited environmental nutrients, such as iron, carbon, and nitrogen sources, thereby restricting its growth and biofilm formation. Iron competition [98,99]: The growth and virulence expression of *S. aureus* are highly dependent on iron. Probiotics (e.g., *Bifidobacterium* spp.) can efficiently sequester iron, reducing the availability of free iron in the environment. Carbon source competition [100]: Certain probiotic strains (e.g., *Lactobacillus* L8) consume key carbon sources (e.g., fucose) required for MRSA growth, thereby limiting its proliferation. Moreover, studies indicate that the metabolic versatility of probiotics—such as their ability to utilize diverse carbon and nitrogen sources—confers a competitive advantage over *S. aureus* in nutrient-limited environments [101].

#### 4.1.2. Production of Antimicrobial Metabolites

Probiotics directly inhibit the growth, adhesion, and biofilm formation of *S. aureus* through the synthesis and secretion of a variety of antimicrobial metabolites.

1. Bacteriocin

Bacteriocins are a class of ribosomally synthesized, target-specific antimicrobial peptides produced by bacteria. They primarily function by disrupting bacterial membrane integrity (e.g., through pore formation leading to ion leakage) [102] or by inhibiting cell wall synthesis [103]. Lactic acid bacteria (LAB), particularly species of *Lactobacillus* and *Enterococcus*, are well-recognized producers of bacteriocins with demonstrated activity against *S. aureus* [104]. For instance, the widely studied lantibiotic nisin binds to the cell wall precursor lipid II [104,105], while enterocins such as DD28/DD93 disrupt initial adhesion and effectively inhibit MRSA biofilm formation [106]. Recent work has also identified bacteriocin-like inhibitory substances (BLIS) from fermented foods, highlighting these environments as valuable sources for discovering novel bacteriocins [107].

2. Organic acid

Organic acids (e.g., lactic acid, acetic acid, and other short-chain fatty acids) produced by probiotics, particularly lactic acid bacteria and bifidobacteria, exert antibacterial effects through three main mechanisms. First, they lower the local pH, creating an acidic environment unfavorable for the growth of *S. aureus*, leading to growth arrest or even cell death [108]. Second, the stability of the biofilm extracellular polymeric substance (EPS) matrix is reduced under acidic conditions, making it more susceptible to degradation or penetration [109]. Finally, certain organic acids such as propionic acid can downregulate the expression of *S. aureus* virulence genes (e.g., those encoding α-hemolysin and enterotoxins) [110], thereby suppressing its pathogenicity.

3. Biosurfactants and Lipopeptides

Biosurfactants are amphiphilic molecules produced by various probiotics. They function by disrupting biofilms, preventing bacterial adhesion, and acting as “penetration enhancers” that facilitate the entry of other antimicrobial agents—such as bacteriocins, organic acids, and hydrogen peroxide—into bacterial cells [111,112]. Among these, lipopeptides (e.g., fengycin produced by *Bacillus subtilis* and LamD558 produced by *Lactiplantibacillus plantarum*) play a prominent role. They directly suppress virulence gene expression (e.g., of α-hemolysin and phenol-soluble modulins) and inhibit biofilm formation in *S. aureus*, primarily by interfering with its Agr quorum-sensing system [113,114].

4. Hydrogen peroxide (H_2_O_2_)

Hydrogen peroxide (H_2_O_2_) exerts antibacterial effects by inducing oxidative damage to the cell membrane and DNA of *S. aureus* [115,116]. Although *S. aureus* produces catalase for self-protection, the high concentrations of H_2_O_2_ generated locally by probiotics can overwhelm this defense mechanism [117]. This allows probiotics to effectively suppress *S. aureus* growth, as demonstrated in contexts such as the vaginal microenvironment for infection prevention [118,119].

#### 4.1.3. Immunomodulatory Mechanisms

Probiotics play a significant role in combating *S. aureus* infections by modulating the host immune system, primarily through the following two mechanisms.

1. Enhancement of Immune Defense and Barrier Function

Probiotics can activate both innate and adaptive immunity. Polysaccharide components (e.g., lipopolysaccharide-like structures) within probiotic biofilms stimulate immune cells such as macrophages [120] and promote the secretion of effector molecules, including immunoglobulin A (IgA) [121] and interleukin-6 (IL-6) [122], thereby enhancing the clearance of pathogens. Meanwhile, probiotics significantly strengthen physical barrier functions [123] by, for example, consolidating the integrity of the intestinal mucus layer [124] and upregulating the expression of tight-junction proteins (e.g., ZO-1, Claudin), thereby reinforcing the epithelial barrier and effectively preventing the colonization and invasion of *S. aureus* [125,126].

2. Cytokine Regulation

Probiotics modulate the host inflammatory response. Studies have shown that specific strains (e.g., *Bifidobacterium* and *Lactiplantibacillus plantarum*) can suppress the production of various pro-inflammatory cytokines (such as TNF-α, IL-6, and IFN-γ) induced by *S. aureus* infection, while promoting the expression of the anti-inflammatory cytokine IL-10 [127,128]. This immunomodulatory activity helps mitigate excessive inflammation, thereby facilitating pathogen clearance without causing collateral tissue damage and promoting recovery from infection.

#### 4.1.4. Summary

As shown above, probiotics inhibit *S. aureus* through a multi-pronged ecological strategy encompassing competitive exclusion (for adhesion sites and nutrients), production of diverse antimicrobial metabolites (e.g., bacteriocins, organic acids, biosurfactants, H_2_O_2_), and modulation of host immunity. This indirect yet broad-spectrum mode of action highlights probiotics’ potential as a safe and holistic anti-infective approach, particularly for mucosal colonization and superficial infections.

### 4.2. Probiotics for the Therapy of S. aureus Infections

Probiotics demonstrate promising potential for the therapy of *S. aureus* infections across multiple clinical scenarios, as outlined below.

#### 4.2.1. Skin and Soft Tissue Infections (SSTIs)

Skin and soft tissue infections (SSTIs) are among the most common diseases caused by *S. aureus*, encompassing a wide spectrum of clinical presentations ranging from superficial conditions such as folliculitis and impetigo to deeper infections, including abscesses and cellulitis [129]. In the prevention and therapy of SSTIs, probiotics administered either topically or systemically have shown promising potential. Studies [130] indicate that selected probiotic strains (e.g., *Streptococcus salivarius* K12 and M18 and *Lactiplantibacillus plantarum* 8P-A3) exhibit significant broad-spectrum antimicrobial activity against various common skin pathogens in vitro. Among them, *L. plantarum* 8P-A3 significantly reduces skin colonization by *S. aureus* and effectively prevents infection in wound models. In contrast, the inhibitory effects of strains M18 and K12 are relatively weaker, which may be attributed to the specificity of the bacteriocin spectra produced by different strains [131]. Furthermore, safety assessments have confirmed that the genome of *L. plantarum* 8P-A3 does not contain transferable antibiotic resistance genes, meeting the safety criteria for clinical application of probiotics [132].

#### 4.2.2. Chronic Wound Infection and Healing

For difficult-to-heal infections such as diabetic chronic wounds—where *S. aureus* (including MRSA) is a predominant pathogen [133]—topical application of probiotic preparations has shown promising therapeutic outcomes. The cell-free supernatant (CFS) of *Lactiplantibacillus plantarum* MTCC 2621, formulated into a gel, effectively promoted wound contraction and tissue regeneration in animal models, with efficacy comparable to that of povidone-iodine [134]. It is worth noting that in wound healing, the concentration of CFS in the gel influenced both healing rate and inflammatory modulation; higher concentrations (50–100% CFS) produced significant effects, although higher concentrations and long-term toxicity were not assessed (the study duration was limited to 21 days). A protein fraction derived from another strain, *L. plantarum* USM8613 [135], accelerated healing by directly inhibiting pathogens while upregulating host β-defensin expression and modulating the balance of wound inflammatory factors (e.g., IL-4, IL-6). Compared to the earlier Lp2621 study, which used only a murine model, the USM8613 protein fraction proved effective in both porcine and rat models, suggesting that its efficacy may be translatable across species.

#### 4.2.3. Nasal and Gastrointestinal Decolonization

Nasal colonization is a significant source of healthcare-associated infections, particularly among high-risk populations (e.g., surgical patients, ICU patients), where it can readily lead to active infections. Intestinal colonization by *S. aureus* may facilitate transmission via feces or intestinal translocation, potentially causing skin and soft tissue infections, surgical site infections, or bloodstream infections [136]. A randomized controlled trial [137] demonstrated that oral administration of a specific probiotic strain (*Bacillus subtilis* MB40) effectively reduced *S. aureus* colonization in humans. After 30 days of intervention, the loads of *S. aureus* in the intestine and nasal cavity decreased by an average of 96.8% and 65.4%, respectively. This probiotic exerts its specific inhibitory effect by producing fengycins, which interfere with the quorum-sensing system of *S. aureus*, and has shown a favorable safety profile. Although participant heterogeneity was observed, the probiotic consistently reduced bacterial loads across all subgroups. Due to its transient colonization in the body, continuous supplementation is required to maintain the preventive effect, making it suitable for long-term prophylaxis in high-risk populations.

#### 4.2.4. Biofilm-Associated Device Infections

Biofilm-associated device infections pose a significant clinical challenge, particularly those caused by *S. aureus* (including MRSA) on the surfaces of orthopedic implants. Such infections are often refractory to therapy and can lead to implant failure, with approximately 74.3% of cases requiring prosthesis removal and multiple revision surgeries [138]. To address this challenge, a study [122] proposed an innovative strategy: using an engineered inactivated *Lacticaseibacillus casei* biofilm as an implant coating. This coating serves a dual function: first, it exhibits potent eradication activity against MRSA by secreting lactic acid and bacteriocins (achieving 99.98% antibacterial efficacy in vitro and 98.1% clearance in vivo); second, its polysaccharide components activate macrophages, promoting the secretion of osteogenic factors and thereby significantly enhancing osseointegration (increasing the bone volume ratio around the implant to 26.89%). Notably, the antibacterial effectiveness of the biofilm coating correlates positively with its maturity (culture time), with optimal results observed after three days of culture. This inactivated coating avoids the risks associated with live bacteria and demonstrates good biocompatibility, offering a novel direction for developing implants that combine anti-infective and pro-osteogenic functions.

#### 4.2.5. Urinary Tract Infections (UTIs) and Vaginitis in Women

Probiotics show potential in the prevention and therapy of urinary tract infections (UTIs) and vaginitis in women, though their efficacy varies depending on the specific condition and route of administration. Regarding the prevention of recurrent UTIs, the existing evidence remains inconsistent: a 2024 study indicated that probiotics, especially when applied intravaginally, can reduce recurrence rates [139], whereas an earlier meta-analysis did not find clear benefits [140]. In contrast, for bacterial vaginosis and vulvovaginal candidiasis, multiple studies consistently suggest that specific probiotic strains (e.g., *lactobacilli*), administered either vaginally or orally, can help restore microbial balance, alleviate inflammation, and reduce recurrence [141,142,143]. Overall, probiotics, particularly when applied topically, represent a promising adjunctive therapeutic option, though their clinical application still requires further optimization of strains and protocols for more definitive recommendations.

#### 4.2.6. Summary

We can see that probiotics demonstrate promising, albeit context-dependent, therapeutic potential across a spectrum of *S. aureus* infections, including SSTIs, chronic wounds, gastrointestinal/nasal decolonization, biofilm-associated device infections, and certain urogenital conditions. Their efficacy is influenced by strain specificity, formulation, delivery route, and host factors. While safety is generally favorable, the translation of these promising results into standardized clinical practice requires further optimization and high-quality evidence.

### 4.3. Challenges in the Clinical Application of Probiotics

The process of converting probiotics from laboratory research to clinical application faces multiple and interwoven challenges. One of the main obstacles is the lack of standardized clinical guidelines [144]. The absence of a standardized treatment process leads to significant differences in the therapeutic efficacy among doctors. This disparity fundamentally stems from the heterogeneity and limitations of clinical research evidence, as discussed in the previous section on clinical application. The efficacy is influenced by multiple factors, including strain specificity, metabolite concentration, host heterogeneity, treatment protocols, and animal models. This makes it difficult to compare and translate the results [145].

A more profound obstacle lies in the conflict between individualized treatment outcomes and universal methods. The initial state of the host’s gut microbiota, genetic background, and dietary habits significantly affect the colonization and efficacy of probiotics, which means that the same strain may have different therapeutic effects on different individuals [146]. Moreover, there are still many issues regarding the quality, safety, and regulation of probiotics. Problems such as insufficient live cell counts, inaccurate strain identification, and the lack of long-term safety data are widespread [147]. Finally, in response to the challenge of intestinal colonization, new delivery technologies need to be developed to ensure that sufficient viable bacteria can survive in the gastrointestinal fluids and successfully colonize the intestinal tract [148].

In summary, these challenges are interrelated and collectively constitute the critical barriers that must be overcome to achieve precise, effective, and safe clinical application of probiotics.

## 5. Advances in Research on Bacteriophage Against *S. aureus*

Similar to probiotics, bacteriophages exert multi-faceted inhibitory effects against *S. aureus* and have demonstrated favorable therapeutic efficacy across a broad spectrum of clinical settings.

### 5.1. Mechanisms of Bacteriophage-Mediated Inhibition of S. aureus

**Figure 3:**
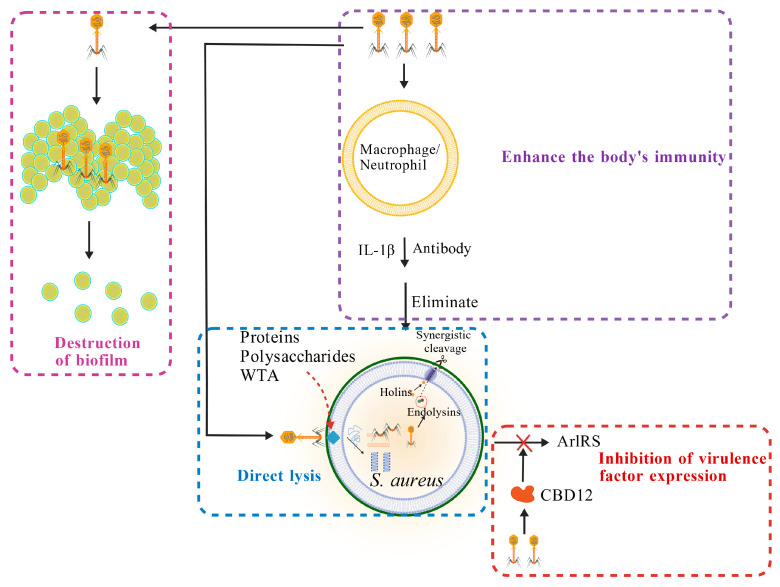
Mechanisms of Phage-Mediated Antagonism Against *S. aureus.* (Created with BioGDP.com [27]). Notes: This figure illustrates four primary mechanisms of *S. aureus* elimination: **Direct lysis mechanism**: bacteriophages directly lyse *S. aureus* through adsorption, genome injection, synthesis, assembly, and release. **Inhibition of** *S. aureus* **virulence factor expression**: for example, the recombinant lysin CBD12 can suppress the expression of virulence factors in *S. aureus*. **Activation of innate immunity**: bacteriophages enhance the host’s immune capacity against *S. aureus* by activating macrophages or neutrophils, which in turn secrete the inflammatory cytokine IL-1β and related antibodies. **Disruption of** *S. aureus* **biofilms**: bacteriophages penetrate and disassemble biofilms through successive rounds of bacterial lysis within the biofilm structure.

The mechanisms by which bacteriophages inhibit *S. aureus* mainly include four aspects, as shown in Figure 3.

#### 5.1.1. Direct Lysis

Bacteriophage infection begins with the specific recognition and adsorption of tail proteins to receptors on the *S. aureus* cell wall, such as proteins, polysaccharides, or wall teichoic acids (WTA) [149]. Following adsorption, the phage injects its genetic material (DNA or RNA) through its tail apparatus into the bacterial cell, thereby hijacking the host’s metabolic machinery to initiate replication [150]. The final step of the bacteriophage lytic cycle involves the synergistic disruption of the bacterial envelope from the inside by a set of lytic proteins, resulting in bacterial lysis and the release of progeny phages. This process is precisely accomplished through the coordinated action of two classes of functional proteins. Holins: These proteins accumulate on the cytoplasmic membrane and form pores, creating channels for endolysins [151]. Endolysins: As peptidoglycan hydrolases, they reach the cell wall via the pores formed by holins and specifically cleave the peptidoglycan structure. This cleavage impairs the integrity of the cell wall. Since *S. aureus* maintains a high internal turgor pressure, the weakened cell wall triggers rapid osmotic lysis, thereby completing the entire lytic process [152].

#### 5.1.2. Biofilm Disruption Strategies

Bacteriophages can disrupt *S. aureus* biofilms through multiple mechanisms [153]. Phage-mediated biofilm disruption primarily involves the following pathways: (1) Stepwise penetration: Progeny phages progressively infiltrate the biofilm interior via repeated cycles of infection, lysis, and reinfection [154]; (2) Matrix degradation: Released bacterial contents (e.g., DNases, proteases) degrade extracellular polymeric substances (EPS), creating channels for phage diffusion [154]; (3) Cascade collapse: Lysis of individual cells may trigger a chain of lysis in surrounding bacteria, leading to a “domino effect”-like structural collapse of the biofilm [155]; (4) Enzymatic synergy: Phage-encoded endolysins (which often exhibit weak polysaccharide-hydrolyzing activity) [156] and depolymerases directly degrade biofilm matrix components [157]. However, it should be noted that certain phages may inadvertently enhance biofilm formation under stress conditions, highlighting the need to combine biofilm-specific assays in phage screening [158].

#### 5.1.3. Immunomodulation

Bacteriophages not only directly lyse bacteria but also modulate host immunity through multiple pathways, synergistically enhancing the clearance of *S. aureus*. The principal mechanisms include: (1) Innate immune activation: They activate macrophages [159] and guide neutrophil migration [149], thereby enhancing the phagocytic and clearance capacity of immune cells against pathogens. (2) Inflammatory balance modulation: Phages can induce the release of pro-inflammatory cytokines (e.g., IL-1β) to restrict bacterial spread [160], while simultaneously promoting the expression of anti-inflammatory cytokines (e.g., IL-10) to prevent excessive inflammation [161]. (3) Potential influence on adaptive immunity: Structural proteins of phages, such as endolysins, may act as antigens and potentially elicit specific antibody responses [162]. (4) Release of immune-stimulatory substances: Bacterial components released during phage-mediated lysis can further activate immune responses [159].

#### 5.1.4. Inhibition of Virulence Factor Expression

Certain phage-derived proteins can suppress bacterial growth through non-lytic mechanisms, offering a potentially safer therapeutic approach. These secreted proteins may mimic toxin–antitoxin systems, inducing a “dormant state” in bacteria and thereby inhibiting the expression of multiple genes, including those encoding virulence factors [163]. For example, infection by some phages (e.g., phiAGO1.3 of the family Podoviridae) can lead to functional loss of virulence-associated genes, such as the ArlRS two-component system, resulting in attenuated bacterial pathogenicity [164]. In contrast to conventional endolysins, which lyse bacteria directly and risk triggering massive toxin release and severe inflammatory responses, certain phage-secreted proteins such as V12CBD do not lyse the bacterial cell. Instead, they exert a dual effect by attenuating bacterial virulence while simultaneously enhancing host immunity, thereby supporting a safer and more controlled clearance of infection. This strategy provides a promising new direction for combating drug-resistant pathogens [165].

#### 5.1.5. Summary

In summary, bacteriophages combat *S. aureus* through direct, potent mechanisms such as targeted bacterial lysis and biofilm disruption, alongside more subtle immunomodulatory and virulence-attenuating effects. This combination of precision killing and host response modulation makes phages a uniquely versatile therapeutic tool, capable of addressing both acute infections and complex, biofilm-associated presentations.

### 5.2. Phage Therapy for S. aureus Infections

#### 5.2.1. Wound Infections and Healing

A case report by Fish et al. [166] demonstrated that weekly intralesional injections of the single phage Sb-1 for seven weeks successfully cured a case of diabetic foot osteomyelitis. The patient achieved complete ulcer healing and bone regeneration, with no recurrence over a three-year follow-up period. In another study by Young et al. [167], adjunctive topical phage therapy (using an unnamed anti-*S. aureus* phage) was administered to ten severely affected patients. Results showed improvement in nine cases, with six achieving complete recovery and avoiding amputation, while overall therapy tolerance was favorable. Collectively, these cases indicate that phage therapy can effectively promote wound healing, reduce the risk of amputation, and exhibit a promising safety profile.

#### 5.2.2. Prosthetic Joint Infection

A study demonstrated that intraoperative local injection combined with short-term intravenous infusion of a single phage (SaWIQ0488o1) successfully cured a recurrent MRSA prosthetic joint infection [168]. In contrast, another experiment showed that an initial regimen using a low-concentration phage cocktail (administered for two weeks) failed; however, subsequent therapy with a high-concentration single phage (SaGR51o1), combined with thorough surgical debridement and prolonged intravenous therapy over six weeks, ultimately eradicated an MSSA infection [169].

In summary, phage therapy for prosthetic joint infections should be employed as an adjuvant to surgical intervention. Its success relies on an integrated strategy combining individualized high-dose regimens with combined local–systemic delivery to ensure the thorough eradication of recalcitrant biofilm-associated infections.

#### 5.2.3. Bacteremia

Intravenous administration of bacteriophages has demonstrated favorable safety profiles in the therapy of *S. aureus* bacteremia and may exhibit synergistic effects with antibiotics. In a single-arm clinical trial, Petrovic Fabijan et al. administered the phage cocktail AB-SA01 to 13 critically ill patients with *S. aureus* bacteremia [170]. The results showed clinical improvement in 54% of patients, with no serious adverse events reported during therapy, indicating the safety of intravenous phage therapy. However, the number of relevant clinical studies remains limited, and the efficacy and broader applicability of this therapeutic approach require further investigation.

#### 5.2.4. Pneumonia

Castledine et al. successfully treated a pediatric case of complex MRSA infection using the single phage ISP combined with antibiotics, achieving rapid clearance of bloodstream infection and eventual eradication of the pulmonary infection [171]. In contrast, Zurabov et al. administered a multi-species phage cocktail tailored to the prevalent ICU flora via nebulized inhalation as a 28-day prophylactic regimen. This approach eliminated multidrug-resistant bacteria from the patients’ airways and alleviated pulmonary inflammation without the use of antibiotics [172]. Together, these studies demonstrate that phages can be applied not only to treat pneumonia but also that their therapeutic strategies can be flexibly adapted between “precision targeting” and “broad-spectrum prevention” depending on the clinical context. Moving forward, the key challenge lies in establishing regulatory and production frameworks capable of simultaneously supporting both models.

#### 5.2.5. Summary

In conclusion, phage therapy has shown encouraging efficacy and safety in treating various drug-resistant *S. aureus* infections, including complicated wounds, prosthetic joint infections, bacteremia, and pneumonia. Success often depends on tailored approaches—such as high-dose regimens, combination with surgery or antibiotics, and adaptive delivery routes. These case studies provide a proof-of-concept for phage therapy’s clinical utility while highlighting the necessity for standardized protocols and further controlled trials.

### 5.3. Challenges in Clinical Phage Therapy

As illustrated by the aforementioned clinical cases, phage therapy has demonstrated favorable efficacy and a high safety profile in combating drug-resistant *S. aureus* infections.

However, the translation of phage therapy from laboratory to clinical practice is confronted with a series of interconnected challenges. Firstly, there is the issue of regulation and approval. Currently, there is a lack of clear regulations for bacteriophages globally, which makes it difficult for them to gain clinical approval. The global regulatory framework is also inconsistent [173]. Secondly, there are difficulties in standardization and quality control. As a biological product, there are no unified standards for its production, purification, and potency determination [174]. Then there is the contradiction between research and development and industrialization. Personalized treatment is highly targeted and precise, but the process is complex, costly, and difficult to scale up [173]. The convenient cocktail therapy is not capable of covering all the bacteria and may even lead to the emergence of drug-resistant bacteria [175]. Finally, there is still a certain gap between basic research and practical clinical application. For instance, how to precisely select the bacteria before treatment, how bacteria develop resistance to bacteriophages, and how bacteriophages affect other microbial communities in the human body—these aspects still require further exploration [176].

These intertwined challenges collectively form the systemic barriers that must be overcome to achieve the safe, effective, and accessible clinical application of phage therapy.

## 6. A Comparative Overview of Probiotics and Bacteriophages

### 6.1. Comparative Analysis of Antibacterial Mechanisms

Probiotics and bacteriophages represent promising natural and eco-friendly therapeutic strategies for combating drug-resistant *S. aureus*. Comparing their mechanisms of action against *S. aureus* is of significant importance for future mechanistic research and the development of novel antibacterial approaches. Based on existing literature, probiotics primarily exert inhibitory effects through competitive exclusion, production of antimicrobial metabolites (such as those disrupting biofilms, suppressing virulence factor expression, or directly killing bacteria), and immunomodulation. In contrast, bacteriophages mainly rely on the lytic cycle, biofilm disruption mediated by lysins, immunoregulation, and suppression of virulence factor expression via derived proteins.

It is evident that, aside from the first one—which is unique to themselves—the subsequent mechanisms are highly similar: (1) probiotics secrete substances like organic acids to disrupt biofilms and produce lipopeptides to inhibit virulence gene expression; (2) bacteriophages employ lysins to degrade biofilms and utilize derived proteins to suppress virulence genes; and (3) both are capable of modulating the host immune response. This logically leads to a pertinent question worthy of further investigation: if probiotics and bacteriophages are used in combination to synergistically inhibit drug-resistant *S. aureus*, what synergistic mechanisms would likely be exhibited? This represents an area that merits in-depth exploration.

### 6.2. Clinical Application Comparison

Currently, both bacteriophages and probiotics have accumulated a certain number of clinical cases in the therapy of wound infections, yet their applications in other clinical scenarios show limited overlap. For instance, gastrointestinal decolonization of *S. aureus* is currently mainly achieved through probiotics, while future clinical studies could explore the potential of bacteriophages for this indication. On the other hand, bacteriophages can be used to treat bacteremia, whereas probiotics may even induce bacteremia in certain circumstances. This suggests that bacteriophages and probiotics may each correspond to distinct clinical indications. In terms of delivery routes, probiotics are generally limited to topical or oral administration, while bacteriophages can additionally be administered via intravenous injection. It can be seen that bacteriophages have a broader scope of application. However, in the future, combining the ecological modulatory capacity of probiotics with the precision targeting capabilities of bacteriophages may represent an important direction. For example, engineered phages could be utilized to precisely eliminate pathogenic bacteria, thereby creating favorable conditions for probiotic colonization [177].

### 6.3. Comparative Analysis of Clinical Therapeutics Challenges

During the transition from laboratory research to clinical application, both probiotics and bacteriophages encounter a series of complex and interwoven challenges. The following provides a comparative analysis of the two from several core dimensions:

1. The common challenges

First, both are used as biological therapeutic agents. The existing medical product regulatory and evaluation systems are not fully compatible with them, resulting in a lack of recognized standards throughout the production and application processes. Second, both of them are biological preparations of living organisms. Their purity, activity, concentration, and batch consistency cannot be strictly controlled like antibiotics, which directly affects the assessment of efficacy and safety. Finally, the mechanisms of both approaches involve complex interactions between organisms (probiotics–host, bacteriophages–bacteria–host). There are still many issues regarding the translation of in vitro and animal experiment evidence into human clinical applications.

2. The different Challenges

At the therapeutic level, the fundamental contradiction of probiotics lies in the conflict between individualized efficacy and universal methods. Their effectiveness highly depends on the initial microbiota of the host, as well as the genetic and dietary background, and there is a lack of universal treatment plans. However, phage therapy faces the contradiction between personalized precision and large-scale production. At the industrialization level, the main bottlenecks for probiotics lie in strain selection, cultivation stability, and the retention of viable bacteria during storage, while for bacteriophages, the focus is on building a rapid diagnostic-matching platform and optimizing the production process for personalized or cocktail therapies. In terms of safety, more attention is required regarding the long-term safety data of probiotics and their potential disturbances to the host’s complex microbiota, while for bacteriophages, more caution is needed regarding the possibility of carrying toxin genes as viruses, triggering lysogenic conversion, and their long-term effects on microbial ecology.

In conclusion, both require the establishment of regulatory scientific frameworks, quality control standards, and clinical evaluation systems that are adapted to their biological characteristics. At the same time, the development of probiotics needs a deeper understanding of the mechanisms of individualized responses, while phage therapy needs to find a feasible balance between personalized treatment models and industrial production. In the future, the successful transformation of both may rely on the development of companion diagnostic technologies (such as microbiota analysis and rapid pathogen identification), thereby achieving true precision medicine.

## 7. Conclusions and Prospects

This review systematically elucidates the severe challenges currently faced in the clinical management of drug-resistant *S. aureus* infections, with conventional antibiotic therapy increasingly failing in the face of synergistic interactions between multifactorial resistance mechanisms and virulence factors. Against this backdrop, probiotics and bacteriophages have emerged as two promising green biocontrol strategies with significant potential. Probiotics exert their effects through multiple, indirect ecological mechanisms—such as competitive exclusion, secretion of antimicrobial metabolites (e.g., bacteriocins, organic acids), and immunomodulation—demonstrating favorable safety profiles in areas such as skin and mucosal infections and decolonization. In contrast, bacteriophages are characterized by precise and potent mechanisms, including direct bacterial lysis, efficient biofilm disruption, immune modulation, and suppression of virulence expression, and have achieved breakthrough outcomes in deep-seated and refractory infections. While their modes of action differ markedly, they share profound overlaps in areas such as biofilm disruption and host immune regulation, providing a theoretical foundation for developing synergistic therapeutic approaches. However, their clinical translation pathways are fraught with distinct challenges. Both probiotics and phage therapy face challenges of standardization, regulation and quality control when moving towards clinical trials. More specifically, their core challenges differ. For probiotics, the therapeutic effect is highly dependent on individual differences in the host, making it difficult to achieve a universal solution, and the problem of live bacteria colonization needs to be overcome. In contrast, phage therapy struggles to strike a balance between highly personalized precision treatment and large-scale production, while also needing to address scientific issues such as bacterial resistance to phages and their ecological impact.

In the future, research should deepen efforts in three key directions: first, to thoroughly investigate the synergistic mechanisms and optimal sequential protocols for probiotic–phage combination therapies; second, to leverage synthetic biology to develop engineered strains/phages and intelligent delivery systems that enhance therapeutic efficacy and targeting specificity; and third, to actively promote the establishment of standardized quality control systems and regulatory pathways tailored to their biological characteristics, while accumulating high-level evidence through rigorous clinical trials.

It should be noted that although this article strives for a comprehensive review, there are still some limitations: First, although the literature screening covers multiple databases, it may still miss studies that have not been included or those published recently. Second, due to the length and the nature of the review, certain molecular mechanisms (such as the precise interactions between virulence factors and immune cells and the action pathways of phage proteins) have not been explored in depth. Third, the discussion on the emerging challenges that therapies may face in the future (such as the environmental risks of engineered strains or phages and the impact of long-term use on the evolution of the microbiome) is not sufficient.

## Data Availability

No new data were created or analyzed in this study. Data sharing is not applicable to this article.
